# Physical activity as a predictor of activities of daily living in older adults: a longitudinal study in China

**DOI:** 10.3389/fpubh.2024.1444119

**Published:** 2024-10-25

**Authors:** Ling-Ying Wang, Hong-Xiu Chen, Hong Zhu, Zi-Yi Hu, Chun-Fen Zhou, Xiu-Ying Hu

**Affiliations:** ^1^Critical Care Medicine Department, West China Hospital, Sichuan University/West China School of Nursing, Sichuan University, Chengdu, China; ^2^Innovation Center of Nursing Research and Nursing Key Laboratory of Sichuan Province, West China Hospital, Sichuan University/West China School of Nursing, Sichuan University, Chengdu, China; ^3^Nursing Department, West China Hospital, Sichuan University/West China School of Nursing, Sichuan University, Chengdu, China; ^4^Mental Health Center, West China Hospital, Sichuan University/West China School of Nursing, Sichuan University, Chengdu, China

**Keywords:** older adults, activity of daily living, physical activity, nursing, healthy aging

## Abstract

**Objective:**

This study aimed to assess the prevalence of physical activity and its association with the progression of difficulty performing activities of daily living among older adults in China.

**Methods:**

A population-based prospective cohort study based on China Family Panel Studies (CFPS) data was conducted in 2018 and 2020. This study used a logistic model to empirically estimate the effects on daily living activities among older adults.

**Results:**

A total of 2073 older adults aged 60 years and above were included, 78.0% of whom did not exercise. The logistic regression model revealed several predictive factors for activity of daily living decline among older adults. These included residence status (OR = 0.672; 95% CI 0.519–0.869; *p* = 0.002), age (OR = 0.307; 95% CI 0.169–0.557; *p* < 0.001), ethnicity (OR = 0.511; 95% CI 0.338–0.773; *p* = 0.001), education level (OR = 2.180; 95% CI 1.366–3.479; *p* < 0.001), job (OR = 0.601; 95% CI 0.447–0.810; *p* = 0.001), chronic disease (OR = 0.769; 95% CI 0.604–0.978; *p* = 0.032) and physical activity (less: OR = 0.464; 95% CI 0.300–0.720; *p* = 0.001; adequate: OR = 0.512; 95% CI 0.321–0.816; *p* = 0.005).

**Conclusion:**

Our findings indicate that insufficient physical activity is particularly acute among the older adults. Physical activity has emerged as a significant predictor of decreased daily living activities among older adults. Our research underscores that less and adequate physical activity can prevent a reduction in daily living activities, in contrast to a lack of exercise. The most effective threshold for daily exercise frequency is a session per day, while the ideal exercise duration is 15 min. Additionally, the desired intensity for exercise is characterized by rapid breathing and a noticeable heartbeat, accompanied by slight perspiration. Community nurses play a pivotal role in providing health education on daily exercise to the older adults. It is crucial for nurses in community hospitals to closely monitor the daily exercise habits of the older adults, actively disseminate the benefits of exercise, and enhance their current exercise regimens through effective health education, ultimately improving their quality of life.

## 1 Introduction

The UN General Assembly officially designated the period of 2021 to 2030 as the Decade of Healthy Ageing, emphasizing the urgent need for global policymakers to prioritize policies aimed at enhancing the quality of life for older adults, both in the present and in the foreseeable future, in May 2020 (1). Statistics reveal that in 2021, 14.2% of China’s population consisted of individuals aged 65 and above, marking a remarkable doubling of the share of older adults from 7 to 14% over 21 years (2). Given this trend, it is imperative for policymakers in any country with an aging population to implement comprehensive policies that promote healthy aging. If we want our growing older adult population to continue contributing positively to society, optimizing healthy aging for everyone is crucial. Healthy aging is more important for the older population than merely surviving old age. The Chinese Geriatric Association defines healthy aging through five key dimensions: freedom from chronic diseases, absence of cognitive decline, engagement in family and social activities, maintenance of functional abilities, and adoption of a healthy lifestyle (3).

Exercise, also known as physical activity, is a pivotal aspect of healthy aging because it effectively prevents or alleviates various health concerns, including falls, pain, sarcopenia, osteoporosis, and cognitive decline (4). Engaging regularly in moderate-intensity physical activity has been demonstrated to significantly lower the risk of developing significant mobility disabilities (5). Strength training, aerobic exercises, and flexibility training can increase muscle strength and size, leading to improvements in functional capacity and gait speed (6). Over time, exercise has been proven to reduce the incidence of falls by 23% and decrease the proportion of older adults experiencing one or more falls (7). Regular physical activity also aids in relieving osteoarthritic pain and stiffness in the lower extremities while enhancing overall functional capacity (8).

The World Health Organization (WHO) guidelines strongly recommend that older adults engage in 150 min of physical activity per week, with each session lasting at least 10 min (9). In alignment with these recommendations, the Outline of the “Healthy China 2030” Plan has initiated a nationwide fitness campaign, stipulating that more than 40% of urban and rural residents should regularly participate in exercise by 2030 (10, 11). However, domestic economic development and urbanization processes in China have led to a decline in national physical activity levels. Compared to 1991, the average weekly physical activity among adults in China decreased by 32% in 2006 (12). Furthermore, over the past two decades, the oldest Chinese individuals have increasingly adopted sedentary and solitary lifestyles (13).

An investigation of the senior population in China revealed a startling fact: the overall functional disability rate among older adults soared to a high of 41.0%. This trend was particularly pronounced across different age brackets, with 6.9% among those aged 65–79 years, 23.6% among those aged 80–89 years, and a significant 42.7% among centenarians aged 90–99 years (14). A meta-analysis revealed that engaging in moderate physical activities that demand high mental, physical, and social inputs can yield the most substantial benefits in ADL physical performance (15). Previous prospective cohort studies have consistently shown that exercise can effectively minimize the risk of disability among older adults (16–18). However, these studies on exercise and activities of daily living often rely on loosely defined measurements, focusing narrowly on a single or just a few components (17–19). The frequency of activity is one of the most commonly used metrics in these assessments (19–21).

A comprehensive assessment of the three quantitative components of physical activity frequency, duration, and intensity is crucial to obtain a more nuanced understanding. This allows for a more accurate estimation of the overall volume of physical activities. This finding is significant because different degrees of association between each component and activities of daily living may indicate distinct underlying mechanisms (22, 23). With this in mind, this study aimed to assess physical activity from its quantitative components and its association with the progression of difficulty performing activities of daily living among older adults in China.

## 2 Methods

### 2.1 Data sources

The data utilized in this study originated from the China Family Panel Studies, a comprehensive social survey conducted by the Institute of Social Science Survey (ISSS) at Peking University (24). This project spans 25 provinces, municipalities, and autonomous regions, providing broad coverage of the Chinese population. Specifically, we extracted the data employed in this research from the fifth and sixth rounds of the CFPS.

These questions accurately fit the connotation of the dependent and independent variables and met the research needs of physical activity and activities of daily living of older adults. Centering our theme, we narrowed our focus to individuals aged 60 and above within the database, ultimately securing 5,672 valid samples. Furthermore, this study specifically aimed to investigate the impact of physical activity on ADLs in older adults. Therefore, we conducted a rigorous screening process, excluding samples with missing values, to ensure the accuracy and reliability of our analysis. After this thorough selection, the final sample size was 2073.

### 2.2 Data collection

#### 2.2.1 Activity of daily living

Physical health encompasses the capacity to engage in ADLs, which we assessed by determining the respondents’ ability to perform tasks independently, such as basic activity of daily livings (eating, bathing) and instrumental activity of daily livings (going outdoors, engaging in kitchen activities, using transportation, shopping and doing laundry). We categorized this into three-level variables: 1 = complete dependency, 2 = partial independence, and 3 = complete independence (25). At baseline, individuals were classified as having ADL disability if they reported requiring assistance from another person performing at least one ADL. Utilizing the participants’ ADL status at baseline and follow-up, we further grouped them into two categories: (i) the Maintenance group, which comprised individuals who maintained their self-care status or remained disabled throughout the study period, and (ii) the Descending group, encompassing those who were self-sufficient at baseline but became disabled at follow-up or those who were already disabled at baseline but experienced a worsening of their disability (new or increased ADL) at follow-up.

#### 2.2.2 Physical activity

To assess physical activity among older adults, the participants were asked, “How often do you participate in sports, fitness, and leisure activities? (Sports fitness and leisure activities refer to indoor and outdoor physical activities aimed at strengthening the body and mind, excluding cycling and walking with commuting as the sole purpose). Eight response categories regarding frequency were provided (less than once per month on average, more than once a month on average, but less than once a week, 1–2 times per week, 3–4 times per week, 5 or more times per week, once a day, twice or more times a day, none). Participants who responded that they engaged in exercise then self-reported the duration of their exercise sessions (min) and their perceived exertion (compared to not exercising, there was not much change in breathing and heartbeat; rapid breathing and heartbeat, slight sweating; rapid breathing, significantly increased heartbeat, and excessive sweating) as an indicator of the intensity of each activity. A self-reported questionnaire is a dependable approach for evaluating physical activity. Such questionnaires have been determined to be particularly suitable for large-scale, population-based studies (26, 27).

Participants who did not exercise were classified as having no physical activity. Participants who engaged in less exercise (i.e., not sufficient to meet the national recommendation: participating in physical exercise three times or more per week, with each session lasting for 30 min or longer, and the exercise intensity reaching moderate or higher levels each time) were classified as having less physical activity (10, 11).

#### 2.2.3 Covariates

As covariates in our study, we considered various factors that are inherently interconnected and exert a significant influence on both ADLs and physical activities among older adults individuals. These included age, sex, residency, education level, nationality, marital status, chronic diseases, occupation, body mass index (BMI), and religious beliefs (27–30). Education level was categorized based on the respondent’s highest completed level of education. The lower levels encompassed illiterate/semiliterate and primary/junior high school graduates, while the moderate levels included those with high school/junior high school/technical school/vocational high school qualifications. The higher education levels encompass individuals with college, bachelor’s, or master’s degrees. BMI classification followed the Chinese criteria for adults, dividing individuals into four groups: underweight (BMI < 16.5 kg/m^2^), normal weight (BMI 18.5–23.9 kg/m^2^), overweight (BMI 24–27.9 kg/m^2^), and obese (BMI ≥ 28 kg/m^2^) (31).

Furthermore, we categorized participants into three groups based on their marital status at baseline and follow-up: married (consistently married), unmarried (consistently unmarried), and those experiencing a marital status change (married to unmarried or unmarried to married). Similarly, we grouped individuals into three categories based on their job status at baseline and follow-up: those who consistently had a job, those who were consistently unemployed, and those who experienced a job status change (employed to unemployed or unemployed to employed). To enhance the survey process, the project leverages computer-assisted interviewing techniques, streamlining data collection and bolstering the integrity of the information gathered, which in turn minimizes potential biases.

### 2.3 Statistical analysis

Prior to analysis, a meticulous data cleansing and verification process is undertaken to guarantee precision and completeness, vigilantly scrutinizing for any missing data, outliers, or inconsistencies. In the analytical phase, a suite of independent variables including age, sex, residency, education level, nationality, marital status, chronic diseases, occupation, body mass index (BMI), and religious beliefs are meticulously considered to mitigate their influence on physical activity levels and ADLs.

All collected data were categorical variables, and descriptive statistics were utilized to present demographic information in the form of counts and percentages. These counts and percentages were subsequently entered into the Statistical Package for the Social Sciences, version 26.0, and R for Linux 4.0.5 for further analysis. The general characteristics of the older adults, along with physical activity, were considered independent variables and subjected to multivariate analysis. ADL, on the other hand, was designated as the dependent variable. A multivariate logistic regression model was established to assess the relationships between these variables, and the results of this model were reported using odds ratios (ORs) with 95% confidence intervals (CIs) (28).

The model’s performance was evaluated using both discrimination and calibration. Discrimination was quantified through the concordance index (C-index), equivalent to the area under the receiver operating characteristic curve (AUC) in logistic analysis. An AUC closer to 1 indicated superior discriminant ability, while an AUC closer to 0.5 indicated poor discriminant ability (32). A C-index of 0.70 or higher indicates good discrimination (33). The overall discriminative capacity was also expressed using the area under the curve (AUC) of the receiver operating characteristic (ROC) curve. Calibration plots were generated to compare the consistency between the actual and predicted outcomes to assess the calibration of the model. A 45-degree line represented perfect calibration, and adjacency to this line indicated good calibration (34). Receiver Operating Characteristic (ROC) curves were used to determine the optimal cut-off values for exercise frequency, exercise duration, and exercise intensity in predicting the decline in ADL.

## 3 Results

The study included 2073 older adults aged 60 and above. Table 1 summarizes their general demographic characteristics and physical activity levels. Notably, most participants (70.0%) were 60–69 years of age, with males accounting for 51.0% of the sample. Approximately half of the participants (44.9%) were illiterate or semi-literate, and the majority (82.1%) were married. Additionally, most were Han nation (93.8%), and 57.2% resided in rural settings. Regarding health status, 50.1% of the participants maintained a normal weight, and 46.1% were still employed. Only a tiny fraction (6.1%) had religious beliefs, and approximately one-third suffered from chronic diseases. The baseline prevalence of ADL disability was 18.6%. Among the 1,687 participants who were initially free of ADL disability, 298 developed it, and 107 of the 386 participants who already disabled experienced a worsening of their ADL disability during the 2-year follow-up period.

**Table 1:** Characteristics of older adults in our study (*N* = 2073).

Variable	*n*	%
Resident		
Urban	888	42.8
Rural	1,185	57.2
Age (years)		
60–69	1,451	70.0
70–79	563	27.2
≥80	59	2.8
Gender		
Male	1,057	51.0
Female	1,016	49.0
Ethnicity		
Han	1945	93.8
Minority groups	128	6.2
Education		
Illiterate/semi-literate	931	44.9
Low	899	43.4
Middle/High	243	11.7
Marriage		
Married	1701	82.1
Unmarried	280	13.5
Marriage change	92	4.4
BMI		
Underweight	179	8.6
Normal weight	1,038	50.1
Over weight	667	32.2
Obese	189	9.1
Job		
Have a job	956	46.1
None	732	35.3
Change	385	18.6
Religious belief		
Have	127	6.1
None	1946	93.9
Chronic disease		
Have	627	30.2
None	1,446	69.8
Physical activity		
None	1,616	78.0
Less	247	11.9
Adequate	210	10.1
ADL at baseline		
Self-care	1,687	81.4
Disabled	386	18.6

The logistic regression analysis in Table 2 revealed significant associations between the decline in ADLs and various factors among older adults in China. Notably, the following variables emerged as important predictors of ADL decline: residence status (OR = 0.672; 95% CI 0.519–0.869; *p* = 0.002), age (OR = 0.307; 95% CI 0.169–0.557; *p* < 0.001), ethnicity (OR = 0.511; 95% CI 0.338–0.773; *p* = 0.001), education level (OR = 2.180; 95% CI 1.366–3.479; *p* < 0.001), job (OR = 0.601; 95% CI 0.447–0.810; *p* = 0.001), chronic disease (OR = 0.769; 95% CI 0.604–0.978; *p* = 0.032) and physical activity level, where both lower (OR = 0.464; 95% CI 0.300–0.720; *p* = 0.001) and adequate (OR = 0.512; 95% CI 0.321–0.816; *p* = 0.005) levels were found to be associated with an increased risk of ADL decline.

**Table 2:** Logistic regression model of risk factors for ADL decline among older adults in China (*N* = 2,703).

Variable	*P*-value	OR	95% CI	
			Lower	Upper
Resident				
Urban	**0.002**	0.672	0.519	0.869
Rural	Ref.			
Age (years)	0.000			
60–69	**0.000**	0.307	0.169	0.557
70–79	0.016	0.482	0.266	0.871
≥80	Ref.			
Gender				
Male	0.061	0.791	0.619	1.011
Female	Ref.			
Ethnicity				
Han	**0.001**	0.511	0.338	0.773
Minority groups	Ref.			
Education level	0.000			
Illiterate/semiliterate	**0.001**	2.180	1.366	3.479
Low	0.209	1.346	0.847	2.141
Middle/High	Ref.			
Marriage	0.720			
Married	0.483	0.815	0.460	1.443
Unmarried	0.736	0.916	0.548	1.530
Marriage change	Ref.			
BMI	0.609			
Underweight	0.433	0.800	0.458	1.398
Normal weight	0.735	7.074	0.711	1.623
Over weight	0.976	1.007	0.653	1.551
Obese	Ref.			
Job	0.001			
Have a job	**0.001**	0.601	0.447	0.810
Change	0.968	0.993	0.721	1.369
None	Ref.			
Religious belief				
None	0.687	1.104	0.683	1.783
Have	Ref.			
Chronic disease				
None	**0.032**	0.769	0.604	0.978
Have	Ref.			
Physical activity	0.000			
Less	**0.001**	0.464	0.300	0.720
Adequate	**0.005**	0.512	0.321	0.816
None	Ref.			
Constant	0.388	1.615	–	–

Ref, reference. Bold values: *p* < 0.05.

**Figure 1:**
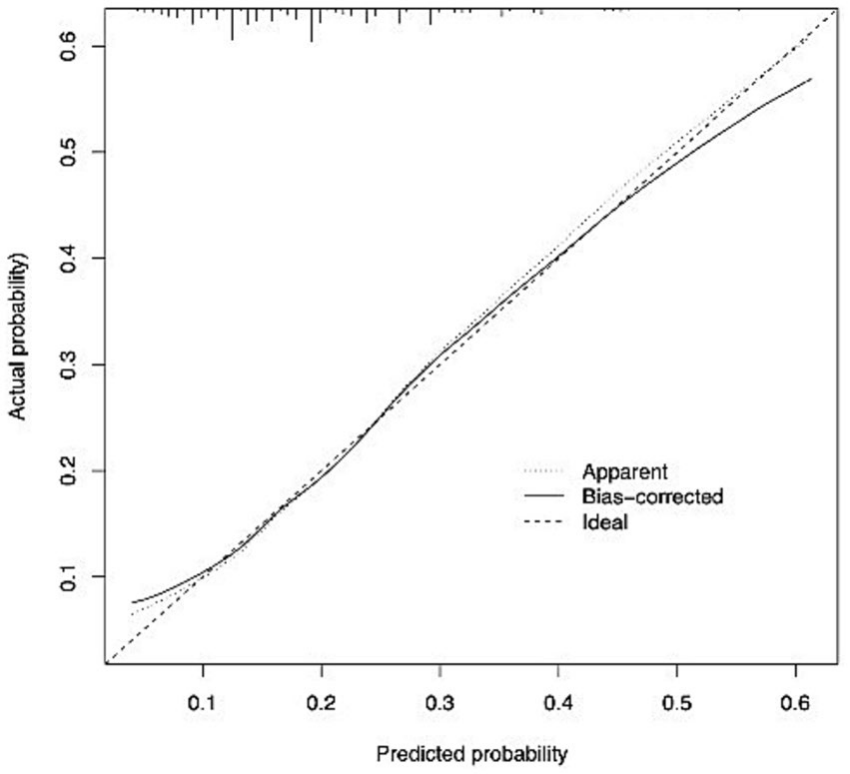
Calibration curve for the model.

**Figure 2:**
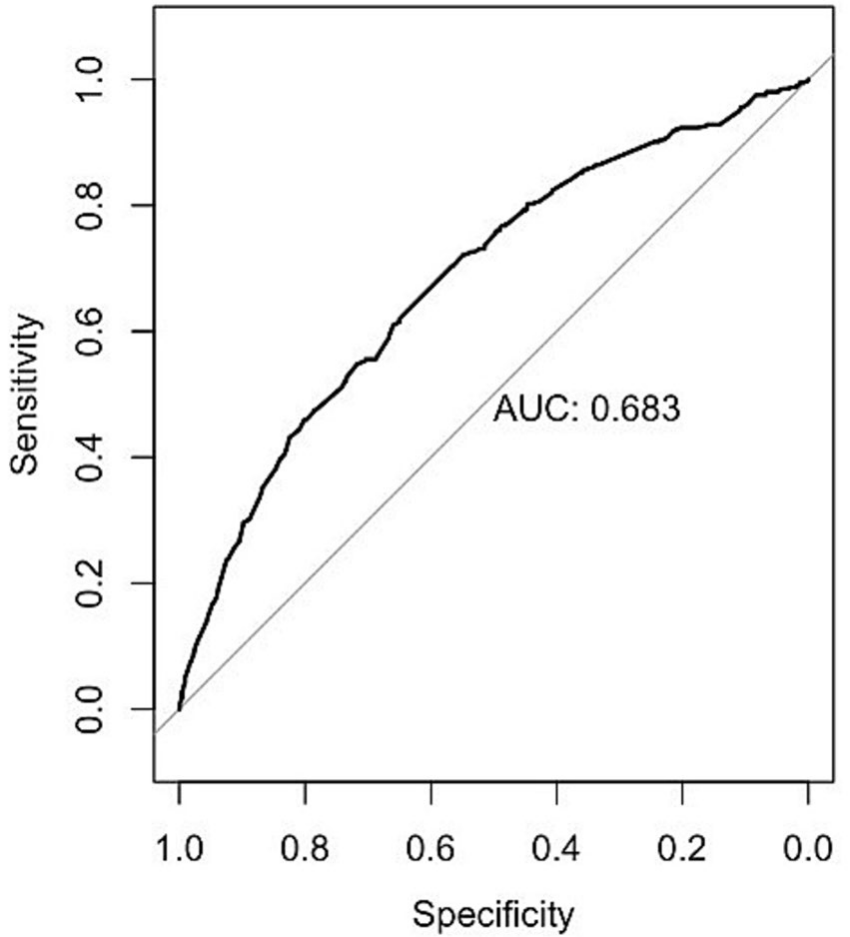
The ROC curve for the model.

The AUC of the model was 0.683, as shown in Figure 1. Additionally, the calibration curve exhibited proximity to the 45-degree line, suggesting excellent calibration, as depicted in Figure 2. Through ROC analysis, we evaluated the predictive ability of exercise frequency, duration, and intensity on the decline in ADL. The results suggested that the optimal cut-off for daily exercise frequency was once per day, the optimal duration was 15 min, and the ideal intensity was characterized by rapid breathing and heartbeat, accompanied by slight sweating.

## 4 Discussion

This longitudinal study revealed that a significant proportion of older adults in China lead inactive lifestyles. Notably, several factors, including residence, age, ethnicity, employment status, chronic disease status, education level, and physical activity, were identified as predictors of ADL decline among this population.

In our study, we observed physical activity in 2073 older adults in China. The Healthy China 2030” Plan encourages adults to participate in physical exercise three times or more per week, with each session lasting for 30 min or longer and the exercise intensity reaching moderate or higher levels each time (10, 11). However, our findings indicate that only 10.1% of the older adults met these criteria, while 78.0% were inactive and 11.9% were less inactive. Previous research has consistently demonstrated that adequate physical activity is crucial for promoting the physical and mental well-being of older adults and enhancing their overall quality of life (35). Unfortunately, physical activity levels tend to decrease with age (36). A global study examining insufficient physical activity pooled data from 358 surveys across 168 countries and estimated global and regional trends in this area from 2001 to 2016. The results indicated that, in 2016, the age-standardized prevalence of insufficient physical activity was 27.5% globally, with women being more affected than men (37). Alarmingly, insufficient physical activity has been ranked as the fourth leading risk factor for global mortality (38) and is estimated to incur a staggering cost of $50 billion annually in global healthcare systems (39). Given these findings, it is imperative to prioritize interventions aimed at increasing physical activity levels among older adults in China.

The overall prevalence of age-adjusted insufficient physical activities among Chinese adults aged 18 years or older rose significantly, increasing from 17.9% in 2010 to 22.3% (with a range of 20.9 to 23.8) in 2018 (40). Notably, the issue of insufficient physical activity was particularly severe among the older adults in our study. The World Health Organization (WHO) published the “Global Recommendations on Physical Activity for Health” as a guiding framework for decision-makers at both the national and regional levels (41). Following this, China also took steps to promote physical activity, publishing “Several Opinions on Accelerating the Development of the Sports Industry and Promoting Sports Consumption” in 2014 (42) and the “Healthy China 2030” Plan outline in 2016 (43). These policies aimed to establish fitness as a national strategy and enhance the physical well-being of the population. Integrating physical activity into various aspects of older adults’ daily lives, including work, transportation, and recreation, is crucial. Nurses working in community hospitals play a pivotal role in this regard. They must pay particular attention to the daily exercise habits of older adults, actively educate them on the benefits of physical activity, and strive to improve their exercise routines through effective health education. This can be achieved through various methods, such as organizing specialized lectures, developing educational brochures or video materials, and leveraging modern social media platforms for widespread promotion.

Our study identified residence, age, ethnicity, education level, occupation, chronic disease status, and physical activity as predictive factors for decreased ADLs among older adults. This finding aligns with previous research, which demonstrated that dependency on ADLs significantly increases with age (44). Alexandre’s study specifically pointed out that among women, only those aged over 80 years exhibited a greater risk of disability. For men, this risk became apparent at 70 and peaked among those aged 80 or older (44). As age advances, the functions of older adults gradually deteriorate, and comorbidities become more prevalent.

Consequently, their ability to perform ADLs rapidly decreases, leading to a more significant proportion of individuals with disabilities. Therefore, age is intricately linked to older adults’ capacity to engage in ADLs. Consistent with our findings, prior research has revealed that rural older adults face a greater risk of ADL limitations than their urban counterparts (45, 46). This could be attributed to the fact that rural and urban areas in China represent distinct social statuses (47). Compared with urban residents, rural residents often engaged in farming tend to have lower educational and income levels (48). Furthermore, rural areas usually need more community infrastructure and have limited access to government-supported public resources or healthcare services (49). This resource-constrained environment further elevates the risk of health issues among older adults (50).

This study revealed a significant association between ethnicity and decreased ADLs among older adults. Specifically, the ADL performance of individuals of Han nationality was superior to that of ethnic minorities, and the risk of ADL decline was significantly lower among the Han population. This observation may be attributed to the fact that many ethnic minority older adults reside in relatively remote or rural regions and often lack pension insurance (51, 52). Consequently, they are unable to access adequate healthcare and endowment security. Furthermore, educational level emerged as a predictive factor for ADL decline, echoing scholars’ assertions that a lower education level is associated with a greater frequency of ADL disability (53–56). Notably, the majority of the older adults in this study were illiterate or semiliterate, which may have influenced their ability to engage in ADLs such as transportation and shopping.

Fujiwara et al. conducted a study involving 6,386 initially non-disabled residents aged 65–84 years in Japan and found that full-time employment can prevent disabilities in older adults, regardless of their frailty status (57). Similarly, Kim’s research revealed that current employment is a reliable predictor of the 5-year risk of severe and persistent ADL disability (58). These findings align with our results, suggesting that work participation among older adults may prevent the onset of all-cause disability. As previously reported, full-time or part-time employment can mitigate the decline in functional abilities (59). This finding supports Jahoda’s latent function theory, which posits that work provides financial and non-financial benefits, including a structured schedule, regular activities, social contact, participation in collective goals, and self-esteem (60). We believe that work is a crucial factor in enabling older adults to maintain a certain level of physical activity, thereby delaying muscle atrophy and enhancing their physical flexibility and coordination. Work, in turn, empowers them to perform better daily self-care tasks, such as dressing, washing, and walking. Work also offers a platform for self-fulfillment, fostering a sense of worth and achievement among older adults. This elevated self-worth greatly enhances their self-confidence and self-esteem, motivating them to take better care of themselves.

Previous studies have shown contradictory evidence on the association between physical activity and ADLs among older adults. For instance, Artaud et al. reported that low to intermediate levels of physical activity were independently associated with an increased risk of disability in a study involving 3,982 older French adults (61). On the other hand, Balzi et al. (62) found that, in a fully adjusted model, a high level of physical activity compared to a sedentary lifestyle was significantly linked to lower incidence rates of both ADL and IADL disability at a 3-year follow-up. Similarly, Wu et al. (63) utilized data from a longitudinal study conducted in four districts of the Taipei metropolitan area. They discovered that the risk of chronic ADL disability was inversely associated with routine exercise among individuals aged 65 and older (63).

Compared with sedentary older people, physically active older adults were more likely to remain living independently (21, 64). Interestingly, our study demonstrated that insufficient and adequate levels of physical activity can prevent a decline in ADLs compared to no exercise. Insufficient level of physical activity can prevent a decline in ADLs may be because (1) Exercise serves as a powerful tool for maintaining or enhancing muscle strength, effectively preventing or alleviating sarcopenia. Exercise not only helps older adults perform various daily tasks, including dressing, washing, and walking, but also boosts their resilience and immunity, thereby minimizing accidents such as falls (18). (2) Exercise has been associated with lower levels of inflammatory markers in older adults, potentially mitigating the adverse effects of inflammation on physical function (65). (3) Exercise offers psychosocial benefits, such as improved walking-related efficacy, which may delay or reduce the emergence of functional limitations (66). Through engaging in group activities, exercising with friends, and other means, older adults can experience a greater sense of social support and emotional care, thereby reducing feelings of loneliness and depression. This positive mindset equips them better to face life challenges and difficulties, ultimately enhancing their self-care abilities. Several proposed mechanisms suggest that adequate levels of physical activity can prevent a decline in ADLs: (1) Regular exercise is linked to a reduced risk of metabolic syndrome, subsequently decreasing the likelihood of developing conditions that could lead to a decline in activities of daily living (67). (2) In general, the more often a person is physically active, the better their physical capability. This is due to adaptations of physiological systems, most notably within the neuromuscular system to coordinate movements, the cardiopulmonary system to more effectively distribute oxygen and nutrients around the body, and metabolic processes particularly those regulating glucose and fatty acid metabolism, which collectively increase overall aerobic power and physical capability (68). (3) Physical activity reduces the risk of developing cardiovascular and metabolic disease through better control of blood pressure, cholesterol and waist circumference in a dose-dependent manner: more activity leads to lower risk of cardiovascular and metabolic disease (69).

Inactivity is the major cause of poor physiological fitness and disease in older age, at least equal to the effects of smoking, drinking excessive alcohol intake and obesity (70, 71). Although PA decreases with age (72), maintenance of PA might be critical for preventing physical decline. “Healthy China Action (2019–2030)” initiative proposes nationwide fitness activities (10), but knowing how to encourage exercise participation at the population-level is challenging because a one-size-fits-all program is not suitable. The intensity of exercise should be modified to appropriately match the individual’s exercise experience and physical capability. It is encouraged for the older adults to choose exercise methods that are suitable for their physical condition and health status according to their capabilities. While emphasizing aerobic exercise, it is also important to focus on muscle strength training and flexibility exercises, and to appropriately engage in balance training to strengthen the musculoskeletal system and prevent falls. It is recommended that the older adults regularly monitor their blood pressure and blood sugar during exercise to adjust the amount of exercise accordingly.

Our study demonstrated that insufficient and adequate levels of physical activity can prevent a decline in ADLs compared to no exercise among older adults. The most effective threshold for daily exercise frequency is a session per day, while the ideal exercise duration is 15 min. Additionally, the desired intensity for exercise is characterized by rapid breathing and a noticeable heartbeat, accompanied by slight perspiration. Enhancing social and environmental support for physical activity can significantly elevate individuals’ engagement in exercise. This includes partnering with others, such as spouses, friends, or colleagues, for mutual motivation. Additionally, strategies that expand access to fitness facilities and redesign public and private spaces, like workplaces, to encourage movement and counteract sedentariness are effective (73). For older adults, sustaining a routine exercise program can be challenging and necessitates ongoing support interventions (73, 74). Research indicates that social support tends to be more effective than targeted cognitive interventions for maintaining exercise adherence (73, 74). For those exercising at home, regular check-ins via telephone to inquire about their progress and offer encouragement can be instrumental in helping individuals stick to their fitness regimen (74, 75). Future research that explores this relationship in different cultural contexts or considers additional factors such as socioeconomic status or access to healthcare facilities.

### 4.1 Strengths and limitations

This study’s key strengths stem from using a vast, nationally representative survey known as the CFPS. Notably, the model employed demonstrated satisfactory discrimination and calibration capabilities. Beyond the information gained through ADL, the CFPS dataset offers detailed and encompassing data on variables of interest and critical covariates. Furthermore, we employed an objective measure of functional ability as an outcome indicator while accounting for numerous confounding factors, including sociodemographics and chronic disease variables.

However, this study has its limitations. First, we did not directly compare our model with prediction models from prior studies. Such a comparison was challenging due to the absence of certain variables required by those models in the CFPS dataset. Second, external validation in a separate cohort needs to be improved and requires further study. It remains to be seen whether the model developed in this study can be universally applied to all older adults in the community. Therefore, additional prospective, multicenter validation studies are necessary to support our findings.

## 5 Conclusion

Our data revealed that insufficient physical activity is particularly pronounced among the older adults. Moreover, physical activity is a significant predictor of ADL decline in older adults. Our findings indicate that inadequate and adequate levels of physical activity can help prevent a decline in ADLs compared to no exercise. The most effective threshold for daily exercise frequency is a session per day, while the ideal exercise duration is 15 min. Additionally, the desired intensity for exercise is characterized by rapid breathing and a noticeable heartbeat, accompanied by slight perspiration. Community nurses play a pivotal role in educating older adults about the importance of daily exercise. Nurses in community hospitals must prioritize the daily exercise habits of older adults, actively impart knowledge on the benefits of exercise, and enhance their current exercise patterns through effective health education, ultimately improving their quality of life.

## Data Availability

The raw data supporting the conclusions of this article will be made available by the authors, without undue reservation.
